# Gigantic, Sixteen‐Pound Carcinoma Ex Pleomorphic Adenoma of the Neck

**DOI:** 10.1002/ccr3.70048

**Published:** 2025-01-16

**Authors:** Andrew J. Rothka, Maria Ferraro, Richard Bavier, Mohamad Saltagi, Sanica Bhele, Neerav Goyal, Guy Slonimsky

**Affiliations:** ^1^ Penn State College of Medicine Hershey Pennsylvania USA; ^2^ Saint Francis University Loretto Pennsylvania USA; ^3^ Department of Otolaryngology – Head and Neck Surgery Penn State Health Milton S. Hershey Medical Center Hershey Pennsylvania USA; ^4^ Department of Pathology Penn State Health Milton S. Hershey Medical Center Hershey Pennsylvania USA

**Keywords:** carcinoma ex pleomorphic adenoma, head and neck cancer, myoepithelial carcinoma ex pleomorphic adenoma, rapid tumor growth, salivary gland malignancy

## Abstract

Carcinoma ex pleomorphic adenomas are rare malignant tumors of the salivary glands, primarily seen in older adults. They originate from benign pleomorphic adenomas, are aggressive, and have poorer prognosis. This case documents an unusual presentation of a massive carcinoma ex pleomorphic adenoma.

## Graphical abstract

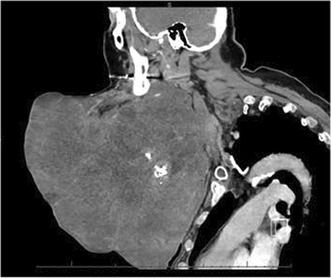

## 1 Introduction

Carcinoma ex pleomorphic adenoma (CXPA) is a rare malignant tumor of the salivary glands that arises from a primary benign pleomorphic adenoma [1, 2, 3, 4, 5, 6, 7]. These tumors account for approximately 5% of all head and neck malignancies [8]. They typically present as a firm mass most commonly found in the parotid gland, with the submandibular gland as the second most common location [1, 6, 9, 10, 11, 12, 13]. The size of the malignant tumor can range from 1 to 25 cm [1, 2, 5, 14]. Suspicion of CXPA often arises when there is a rapid increase in the size of a salivary gland mass or a known pleomorphic adenoma [4, 5]. Patients often present with increasing pain and rapid, visible enlargement of the mass [1]. The gold standard for diagnosing suspected CXPA is fine needle aspiration cytology. However, misdiagnosis can occur, as the malignant component of the tumor can be small, diffuse, and easily missed [1, 4, 15]. Computed tomography (CT) scans help determine the size, degree of calcification, and amount of invasion into the surrounding tissues [15]. Although the risk of developing malignancy from a pleomorphic adenoma is only 1.5% if left untreated for less than 5 years, it increases to 9.5% after 15 years [7, 12, 13, 16]. Surgical intervention is the current treatment recommended for CXPA [1, 4]. The authors present a case of a submandibular gland CXPA insidiously growing to a colossal size.

## 2 Case History/Examination

**FIGURE 1:**
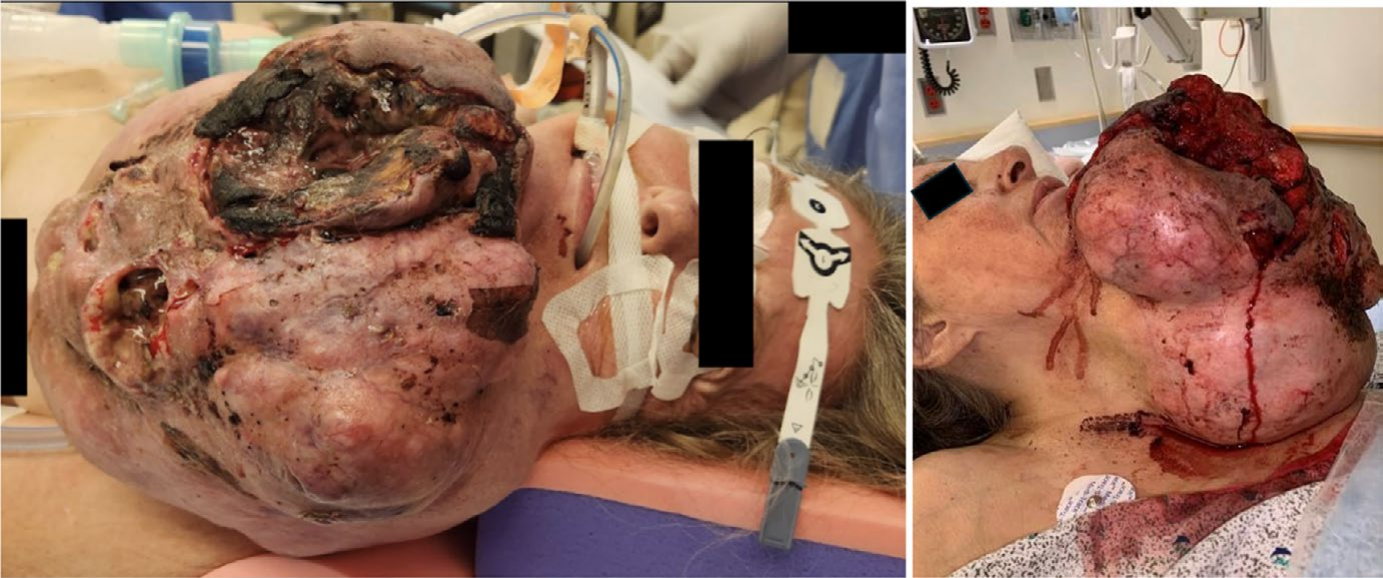
Preoperative images of the neck mass.

**FIGURE 2:**
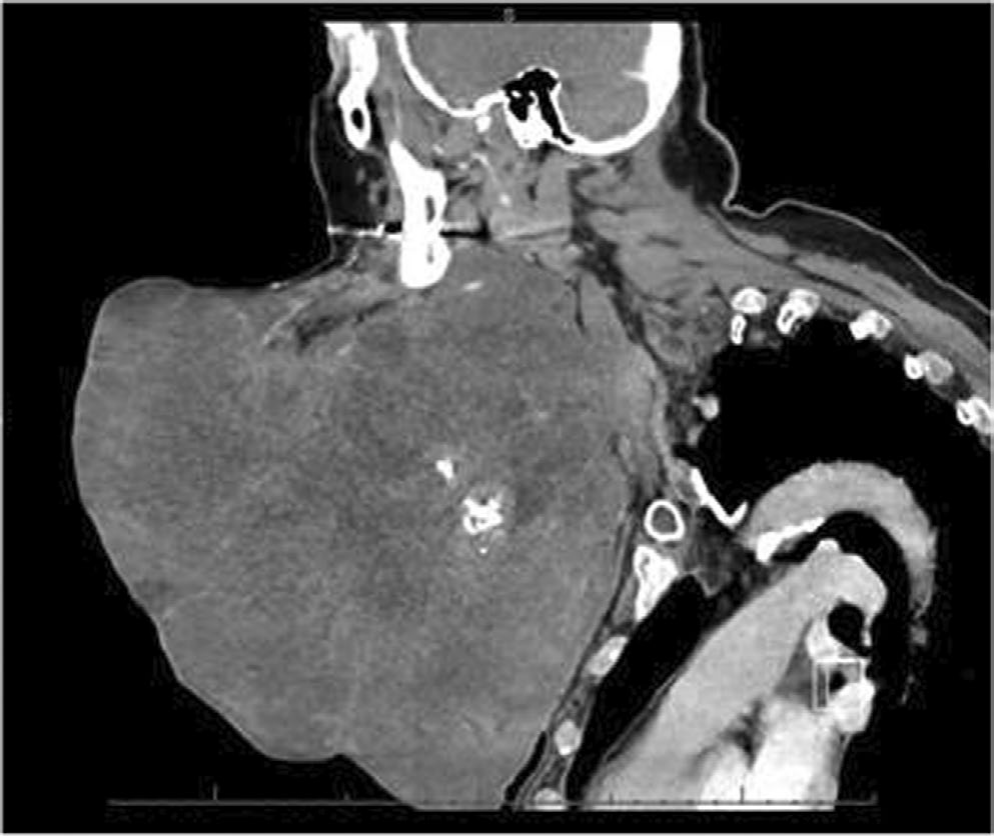
Sagittal CT image of the neck mass at presentation.

A 62‐year‐old female with agoraphobia presented to the Emergency Department (ED) with a hemorrhaging lacerated left‐sided neck mass after a fall. For 9.5 years, the mass was stable at the size of a tennis ball. However, it rapidly grew over 7 months to the size of a basketball (Figure 1). Previous evaluation at an outside institution via CT of the neck with contrast demonstrated a 21.5 × 20.8 × 24 cm heterogeneous lobulated mass with internal foci of necrosis and scattered calcifications (Figure 2). The mass extended from the left mandibular angle down to the anterior chest wall. It was suspected to be a primary salivary gland tumor, possibly a transformed pleomorphic adenoma; however, no biopsy was performed at the outside institution. The patient denied dysphagia, odynophagia, or dyspnea. She endorsed chest, neck, and back pain from the weight of the lesion.

## 3 Investigations and Treatment

On admission, the bleeding was controlled with packing. Due to the significant bleeding encountered after the initial trauma, the patient underwent preoperative embolization of the bilateral facial and left lingual arteries. She underwent excision of the 16‐pound neck mass with wound vacuum dressing placement and bilateral neck dissection in the operating room. The surgical specimen was sent for frozen section evaluation, and the preliminary diagnosis was of a low‐grade spindle cell neoplasm.

## 4 Outcome and Follow‐Up

Postoperatively, the patient was short of breath. Chest X‐ray (CXR) was concerning for mucus plugging in the left lung. She underwent bronchoalveolar lavage, and repeat CXR revealed improved aeration. Three days postoperation, the patient was placed on high‐flow nasal cannula for desaturations and dyspnea. Bedside flexible fiberoptic laryngoscopy demonstrated hypomobility of the right vocal cord. She was eventually intubated due to acute respiratory distress. Four days postoperation, she underwent debridement of a small necrotic skin flap and primary closure. The patient was extubated and underwent bedside right vocal cord medialization 10 days after the mass resection. Fifteen days postresection, the patient was medically cleared and discharged home.

**FIGURE 3:**
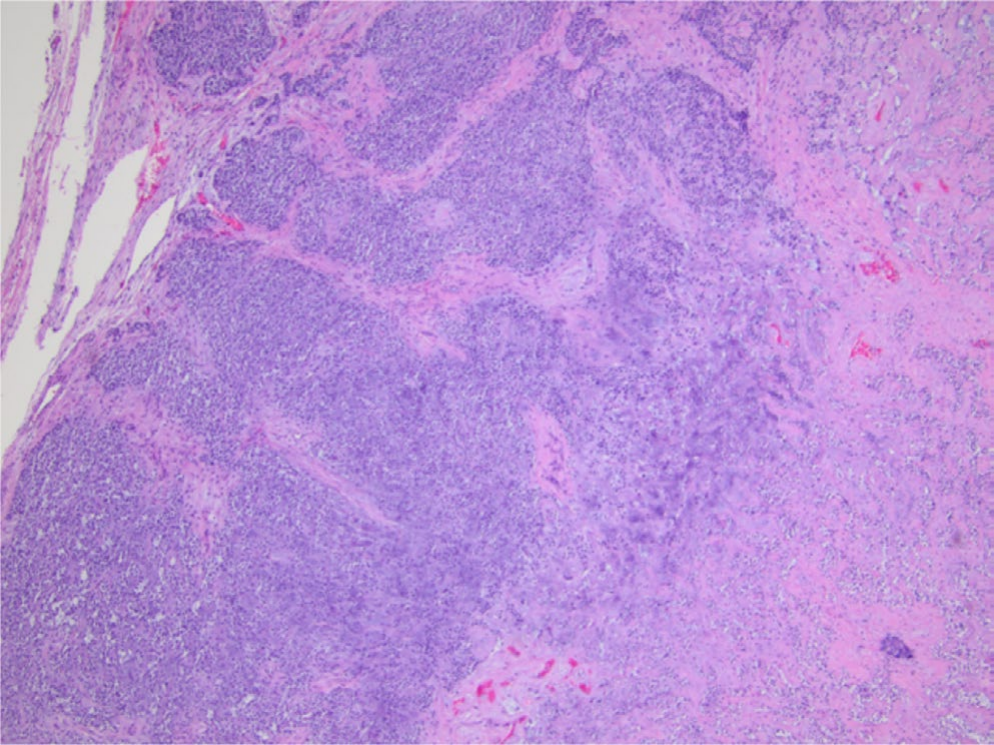
Encapsulated nodular neoplasm with sheets of neoplastic cells with clear cytoplasm and chondromyxoid stroma.

**FIGURE 4:**
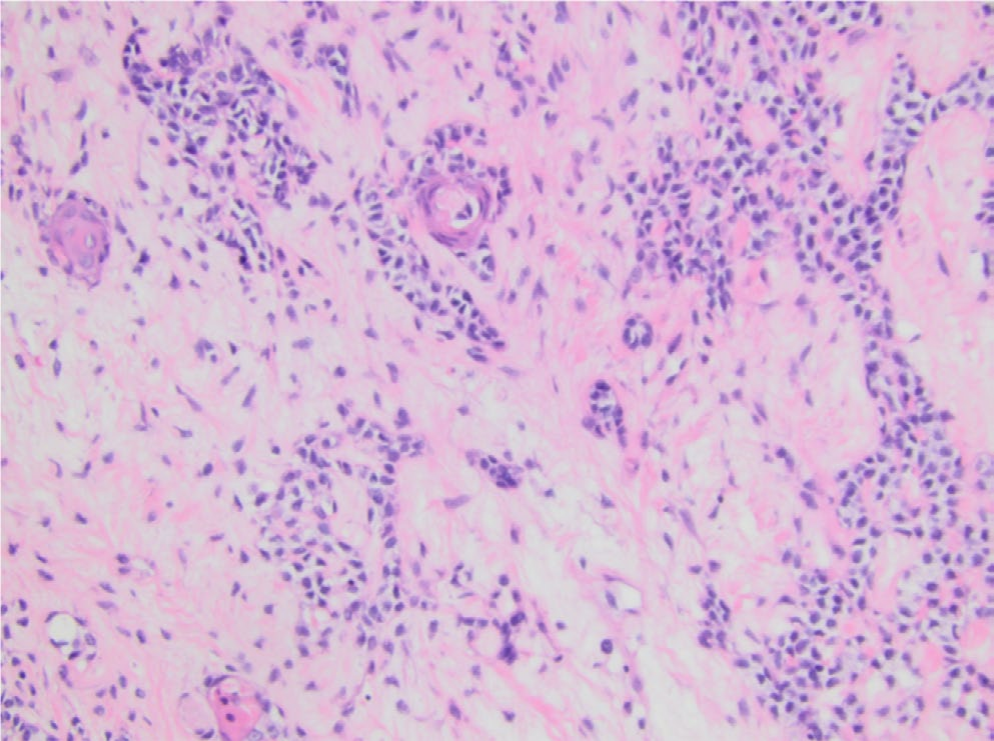
Sheets of neoplastic cells with clear cytoplasm and foci of squamous metaplasia.

**FIGURE 5:**
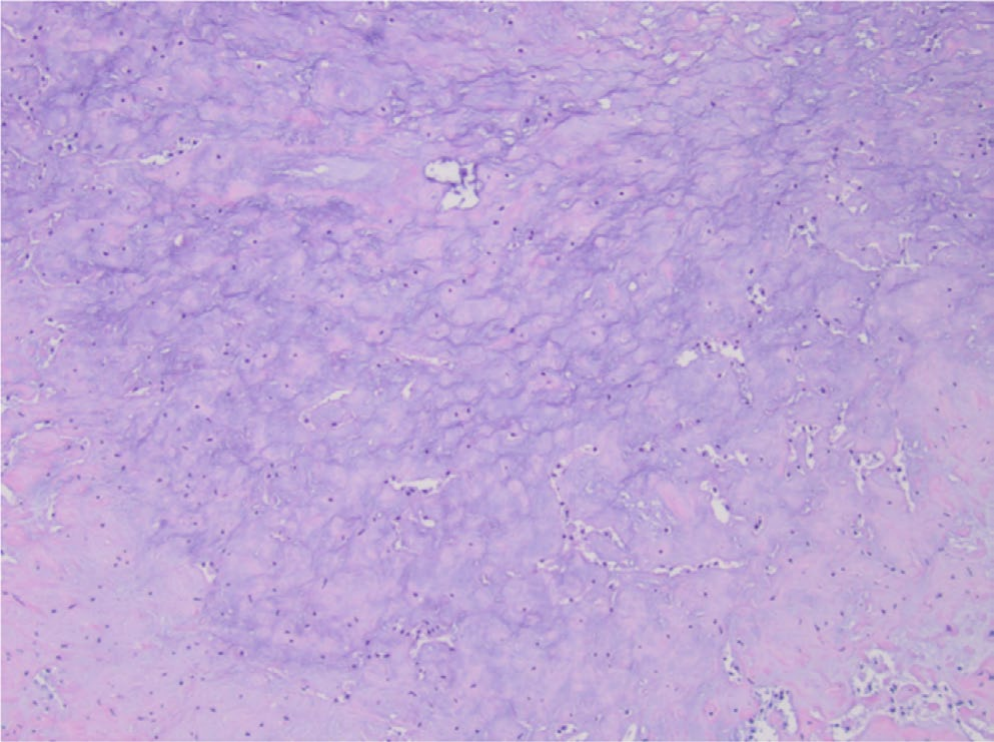
Foci of chondromyxoid stroma of residual pleomorphic adenoma.

**FIGURE 6:**
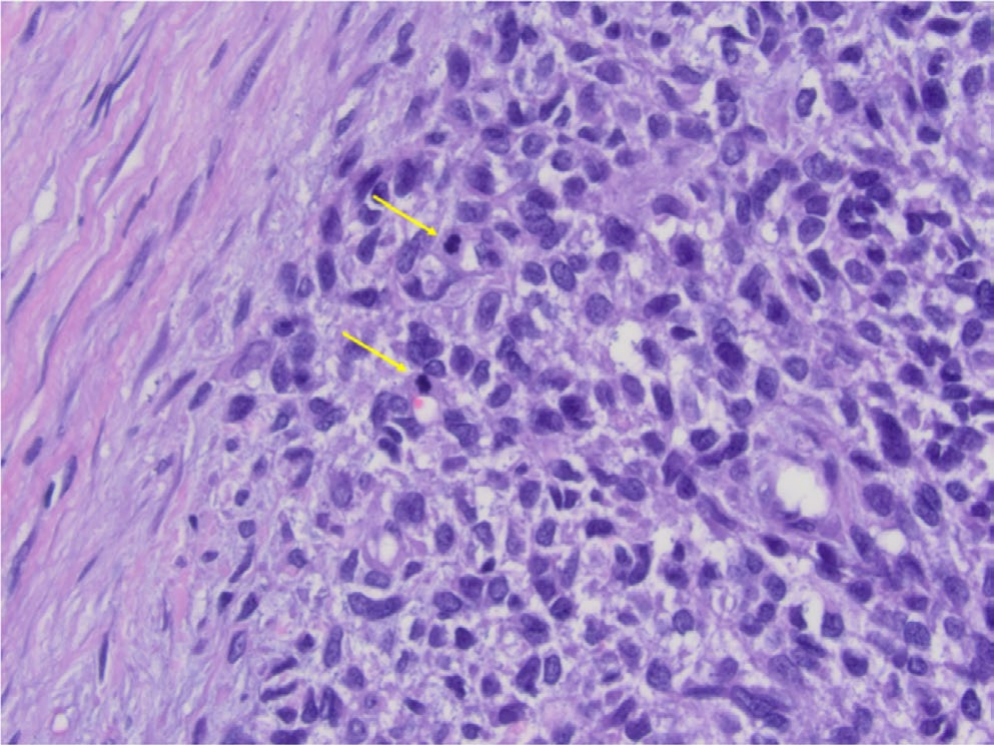
Neoplastic cells with moderate cytologic atypia, readily identifiable mitotic figures in myxoid stroma.

Final histologic sections from the mass displayed a cellular neoplasm with multinodular growth and lobulated borders. The neoplasm showed a transition from a low‐grade morphology to moderately high‐grade morphology in the form of cellular atypia, increased mitotic activity, and tumor necrosis. The benign appearing portion predominantly consisted of sheets of monomorphic spindle to plasmacytoid cells with clear cytoplasm in myxoid to hyalinized collagenous stroma with foci of necrosis, squamous metaplasia, and keratin pearls formation. Foci of chondromyxoid stroma suggestive of residual pleomorphic adenoma were present, but there were no definitive ductal structures identified (Figures 3, 4, 5, 6). Immunohistochemistry demonstrated S100 and p63 expression by neoplastic cells, confirming the myoepithelial origin. The final diagnosis was rendered a myoepithelial carcinoma ex pleomorphic adenoma. Next generation sequencing detected pathogenic *TERT* promoter mutation (c.‐125C>T) and *PIK3R1* mutation (c.1630A>T).

**FIGURE 7:**
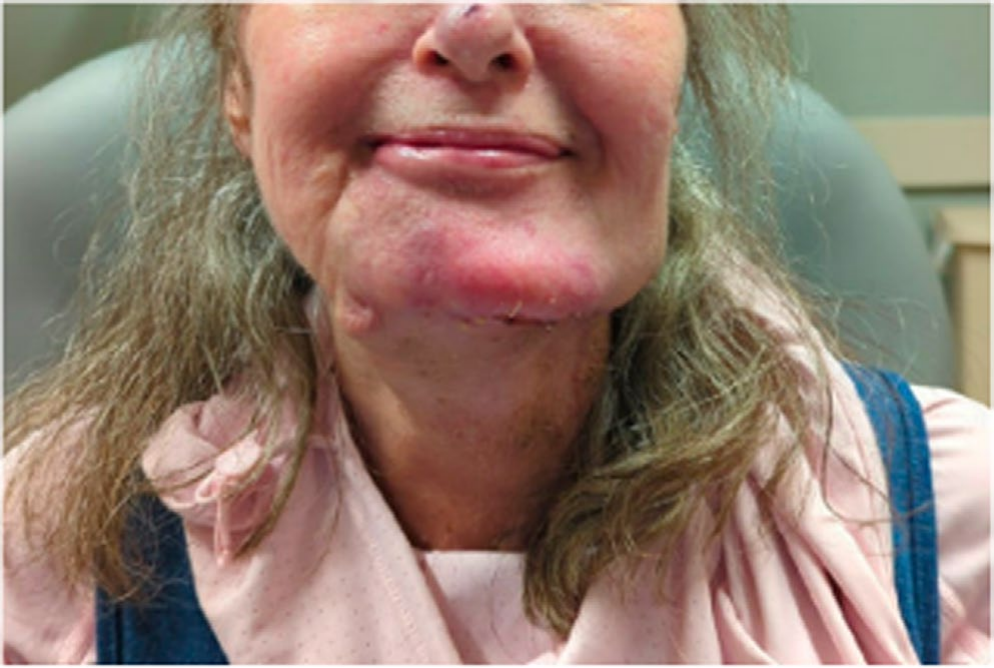
Cosmetic results one month postoperation.

At follow‐up, the patient demonstrated continued improvements in voice quality with recovery of the right vocal cord mobility, and excellent cosmetic results (Figure 7). The patient declined postoperative radiation therapy. Positron emission tomography CT performed 6 weeks postoperation did not demonstrate persistent disease.

## 5 Discussion

CXPAs are rare malignant tumors originating from the epithelial components of a primary or recurrent benign pleomorphic adenoma [1, 2, 3, 15]. To diagnose CXPA, one of two criteria must be present: either a pleomorphic adenoma associated with a carcinoma or a carcinoma that has developed at the site of an existing or recurrent pleomorphic adenoma [9, 17]. The common signs and symptoms of malignancy can include rapid growth, pain, facial nerve palsy, and enlarged lymph nodes [4, 6, 18, 19, 20]. The pathogenesis of CXPA remains poorly understood. However, there are two hypotheses within the literature: first is that the tumor might have been malignant from the start, and second is that malignant transformation occurred within a benign pleomorphic adenoma [5, 9]. Misdiagnosis of CXPA can occur as the malignant component may be small and therefore missed upon histopathological examination. The carcinoma portion may also contain several subtypes of salivary carcinoma making it hard to accurately diagnose and classify [9].

Patients who are diagnosed with noninvasive or minimally invasive CXPA generally have low recurrence rates and no metastases. However, patients with invasive tumors have poorer prognosis [21, 22]. The recurrence rates for invasive CXPA are 23%–50%, and up to 70% of patients can have distant metastases [21, 22]. The current recommendation for treatment is complete surgical resection, which is often followed by radiation therapy, as there is no standard treatment protocol for this type of tumor [1, 4, 23]. The most common area of the body to develop CXPA is the parotid gland, followed by the submandibular glands [1, 6, 9, 10, 11, 12, 13]. Therefore, the most common surgical procedures are parotidectomy and neck dissection [1].

Risk factors associated with the transformation of benign pleomorphic adenomas to carcinomas include smoking history, advanced age, and higher grade tumors [4, 12, 24, 25]. Prognosis of patients with CXPA worsens with disease progression [12]. Therefore, detection, observation, and potential intervention on known pleomorphic adenomas is of high importance. Typically, patients presenting with CXPA had a 10‐ to 15‐year history of a known pleomorphic adenoma, which suddenly underwent rapid growth [7]. This growth usually takes place over an average of 3–6 months [7]. Previous research shows that the average age for patients with CXPA is 55.1–61 years old [5, 15, 18, 26]. The size of CXPA was previously reported to vary from 1 to 26 cm [1, 2, 5, 14, 27, 28]. The patient presented in this case report had a very large CXPA of over 20 cm and weighed 16 pounds. This case demonstrates the surgical management and outcomes of a CXPA that presented as a gigantic neck mass.

The patient presented in this case has a unique subset of biomarkers in tumor pathology. Gene mutations in the PI3K pathway (including *PIK3R1*) are the third most common abnormality in salivary gland tumors. The two most common are the *TP53* gene and mutations in the cyclin pathway [29]. The telomerase reverse transcriptase (TERT) gene has been previously shown to be a more common mutation in oral cavity squamous cell carcinoma when compared to other types of head and neck malignancies [30]. Though previously associated with human papilloma virus (HPV), another study found that ‐124G>A and ‐146G>A mutations in the *TERT* gene significantly affected the development of oral squamous cell carcinoma regardless of HPV‐status [31]. When specifically analyzing salivary gland tumors, *TERT* promoter mutations were not different when comparing malignant and benign tumors, though this promoter and its involvement are rarely investigated [32]. Therefore, the mechanisms for tumor development and transformation into a CXPA are poorly understood.

During the COVID‐19 pandemic, patients with chronic anxiety disorders, including agoraphobia, avoided entering public places [33]. The patient presented here had agoraphobia and stated that she delayed seeking care due to her fear of venturing into public places. Consequentially, the patient did not seek evaluation after the mass began exponentially growing until she sustained a fall and hemorrhage from the lacerated tumor. This case illustrates the importance of facilitating discussions between physicians and patients on timing to seek medical attention and the importance of observing benign neck masses such as pleomorphic adenomas.

## 6 Conclusion

Untreated salivary gland tumors can grow to enormous sizes. Multiple factors can contribute to late presentation, including poor healthcare literacy, lack of access to healthcare, mental health conditions, or fear of surgical treatments. This case highlights the importance of counseling patients about the risks of leaving such tumors untreated and emphasizes potential postoperative complications of gigantic neck mass resections. Further investigation into treatment and prevention modalities may lead to less invasive options for this relatively rare disease.

## Author Contributions

**Andrew J. Rothka:** conceptualization, investigation, writing – original draft, writing – review and editing. **Maria Ferraro:** conceptualization, investigation, writing – original draft, writing – review and editing. **Richard Bavier:** conceptualization, investigation, writing – original draft, writing – review and editing. **Mohamad Saltagi:** conceptualization, investigation, writing – original draft, writing – review and editing. **Sanica Bhele:** conceptualization, investigation, writing – original draft, writing – review and editing. **Neerav Goyal:** conceptualization, investigation, writing – original draft, writing – review and editing. **Guy Slonimsky:** conceptualization, investigation, project administration, writing – original draft, writing – review and editing.

## Ethics Statement

All protected health information was withheld from this piece to ensure patient anonymity.

## Consent

Written informed consent was obtained from the patient to publish this report in accordance with the journal's patient consent policy.

## Conflicts of Interest

The authors declare no conflicts of interest.

## Data Availability

Data sharing is not applicable to this article as no datasets were generated or analyzed during the current study.
